# Analytic modeling of inhomogeneous-resolution maps in cryo-electron microscopy and crystallography

**DOI:** 10.1107/S2052252522008260

**Published:** 2022-09-28

**Authors:** Alexandre Urzhumtsev, Vladimir Y. Lunin

**Affiliations:** a Centre for Integrative Biology, Institute of Genetics and Molecular and Cellular Biology, Illkirch 67404, France; b Département de Physique, Université de Lorraine, Vandoeuvre-lès-Nancy 54506, France; c Institute of Mathematical Problems of Biology RAS, Keldysh Institute of Applied Mathematics of Russian Academy of Sciences, Pushchino 142290, Russian Federation; University of Hamburg, Germany

**Keywords:** real-space refinement, local resolution, interference function, shell decomposition, atomic images

## Abstract

Refinement of macromolecular atomic models versus experimental maps in crystallography and cryo-electron microscopy is a critical step in structure solution. For an appropriate comparison, model maps should mimic the imperfections in the experimental maps, mainly atomic disorder and limited resolution, which are often inhomogeneous over the molecular region. In the suggested method, these model maps are calculated as the sum of atomic contributions expressed through a specifically designed function describing a solitary spherical wave. Thanks to this function, atomic contributions are analytically expressed through their atomic displacement parameter and local resolution, a value now associated with each atom. Such a full analytic dependence of inhomogeneous-resolution map values on model parameters permits the refinement of all of these parameters together.

## Abstract 2

A decomposition of 3D oscillating functions results in analytic expressions for atomic model density maps distorted by inhomogeneous resolution and atomic positional disorder. Such decomposition extends the possibilities of real-space refinement of atomic models.

## 1. Introduction

**Figure 1:**
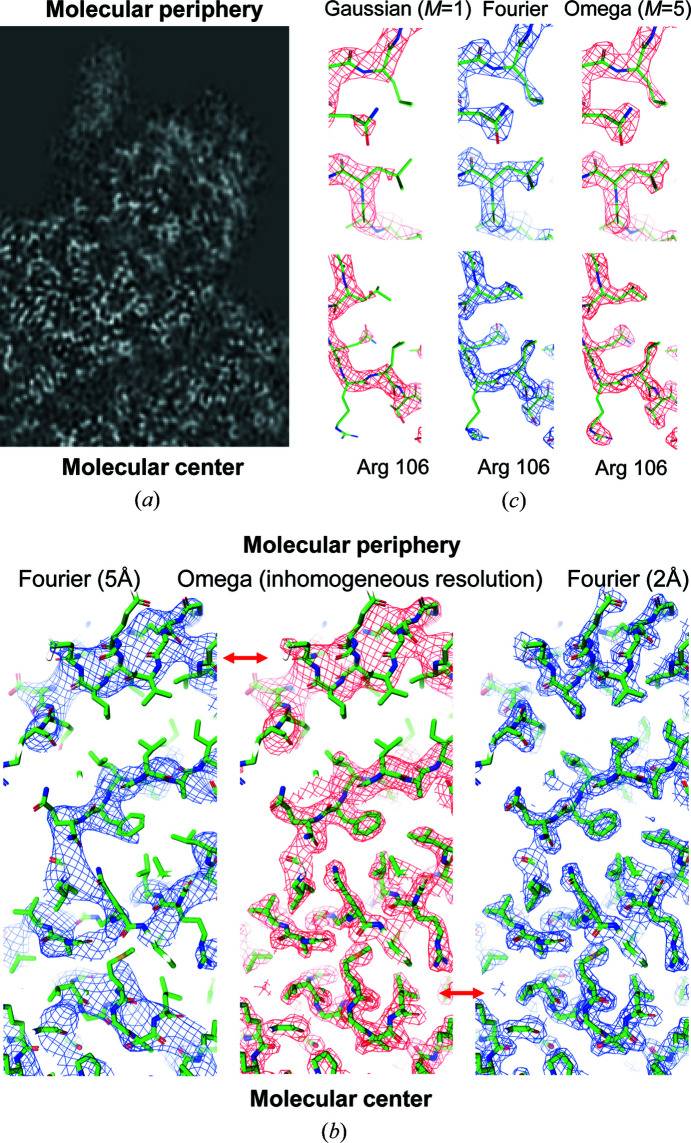
Maps of inhomogeneous resolution. (*a*) A fragment of a cryoEM map (von Loeffelholz *et al.*, 2018 ▸; EMDB 4261) illustrates a decrease of the local resolution from the molecular center to the periphery. (*b*) Test model Ω-map (equation 15) of an inhomogeneous resolution varying from 2 Å in the molecular center to 5 Å at the molecular periphery calculated in a single run by the shell decomposition (middle), and the maps calculated by the Fourier procedure with the resolution of 5 Å (left) and 2 Å (right). Red arrows mark the similarity of different parts of the Ω-map with the Fourier maps of different resolution. (*c*) Fragments of the 2 Å resolution maps contoured to show an equal volume (Urzhumtsev *et al.*, 2014 ▸). The map in the middle was calculated by the standard Fourier procedure. The left-hand map calculated as the sum of the Gaussian approximation, *M* = 1, to the atomic images (no ripples included) reveals the density for some side chains poorly. The right-hand map was calculated by the shell decomposition (equation 15) with *M* = 5 and reproduces the Fourier map correctly. Poor density for the Arg106 residue in the Ω- and Fourier maps can be attributed to large displacement parameters of its atoms. This figure was prepared with *PyMol* (DeLano, 2002 ▸).

Macromolecular atomic models are obtained using maps of electron or nuclear scattering density distributions in macromolecular crystallography, or those of an electrostatic scattering potential in cryo-electron microscopy (cryoEM) and microcrystal electron diffraction. Information extracted from these models depends on the accuracy of their parameters. Owing to the ‘resolution revolution’ in cryoEM (Kühlbrandt, 2014 ▸) and to recent progress in structure prediction of the protein components of macromolecular complexes (Jumper *et al.*, 2021 ▸; Baek *et al.*, 2021 ▸), these maps have become especially important to correct and refine initial atomic models (Diamond, 1971 ▸; Chapman, 1995 ▸; Murshudov, 2016 ▸; Afonine, Poon *et al.*, 2018 ▸; Urzhumtsev & Lunin, 2019 ▸; Yamashita *et al.*, 2021 ▸; Roversi & Tronrud, 2021 ▸; Palmer & Aylett, 2022 ▸) and to validate the results (Helliwell, 2022 ▸). The experimental maps are subject to dynamic and static atomic positional disorder and are available at limited resolution, which often varies from one macromolecular region to another (Cardone *et al.*, 2013 ▸) (Fig. 1 ▸). In order to refine an available model, we define a respective score function by comparing a map calculated from the model with the experimental one and minimize it by varying the model parameters. For an appropriate quantitative comparison, the model map should mimic imperfections in the experimental map. If the model map values are expressed analytically through the model parameters, this could drastically simplify the map calculation and model optimization. Since mathematically the problem and its solution are the same for all methods of structure determination, herein we use the term ‘density’ for both the electrostatic potential and the electron-density distributions.

To mimic the limited resolution and general positional disorder of the experimental map, we start by calculating the exact, theoretical density ρ(**r**) from the model and obtaining its Fourier coefficients. In crystallography, these are known as structure factors. To model the general dynamic and static positional disorder of atoms (or uncertainties in atomic positions) these structure factors are multiplied by the Gaussian function, which is equivalent to blurring the distribution by convolution with the Gaussian function. The required limited-resolution map ρ^*d*^(**r**) is then calculated by the inverse Fourier transform with the set of structure factors cut at the resolution *D* = *d*_high_. Overall, the procedure requires two Fourier transforms, does not provide simple analytic expressions for the derivatives of the score function and significantly complicates obtaining a map of an inhomogeneous resolution, such as those in cryoEM.

Instead, a map can be calculated as the sum of atomic contributions of the respective resolution *D* and the individual degree of disorder characterized by the atomic displacement factor *B* (we call these contributions ‘atomic images’). Both the resolution cutoff and the positional disorder blur atomic images, but the resolution cutoff also results in ripples, spherical shells of locally sign-alternative high density. Several known approximations to atomic images either model only the central peak in the image (*e.g.* Lunin & Urzhumtsev, 1984 ▸; Mooij *et al.*, 2006 ▸; see also Sorzano *et al.*, 2015 ▸, and references therein), interpolate the images precalculated with different *B* values at a given common resolution *D* (DiMaio *et al.*, 2015 ▸), or use a step-function approximation to the atomic scattering function and the integrals of this approximation at a chosen resolution (Chapman, 1995 ▸; Chapman *et al.*, 2013 ▸; Sorzano *et al.*, 2015 ▸).

We suggest a method to calculate model maps at every point, extending the concept of a local resolution (Kucukelbir *et al.*, 2014 ▸; Vilas *et al.*, 2018 ▸; Ramírez-Aportela *et al.*, 2019 ▸) further and presenting the image of every atom *n* in the map with its own resolution *D_n_*, in line with Chapman *et al.* (2013 ▸). We found an explicit expression for the map values using a specially designed analytic function for the atomic position **r**_*n*_, its individual displacement parameter *B_n_* and individual resolution *D_n_*. This expression allows accurate calculation of the inhomogeneous resolution map in a single run, without Fourier transforms. Moreover, it allows simple analytic expressions for the gradient of the score function (Urzhumtseva *et al.*, 2022 ▸) that rules the refinement of the model parameters. Finally, the *D_n_* values can be refined and reported together with other atomic parameters such as coordinates and *B_n_*, and then deposited in databases. The inhomogeneous-resolution maps in cryoEM mean that the suggested method is tailored to this experimental technique while it can also be applied to other types of structural studies in biology and physics.

This article presents the concept of this approach and its basic proofs addressing the direct problem: how to efficiently calculate a map given an atomic model with variable parameters.

## 2. Shell decomposition of oscillating functions

### 2.1. Maps and atomic contributions

**Figure 2:**
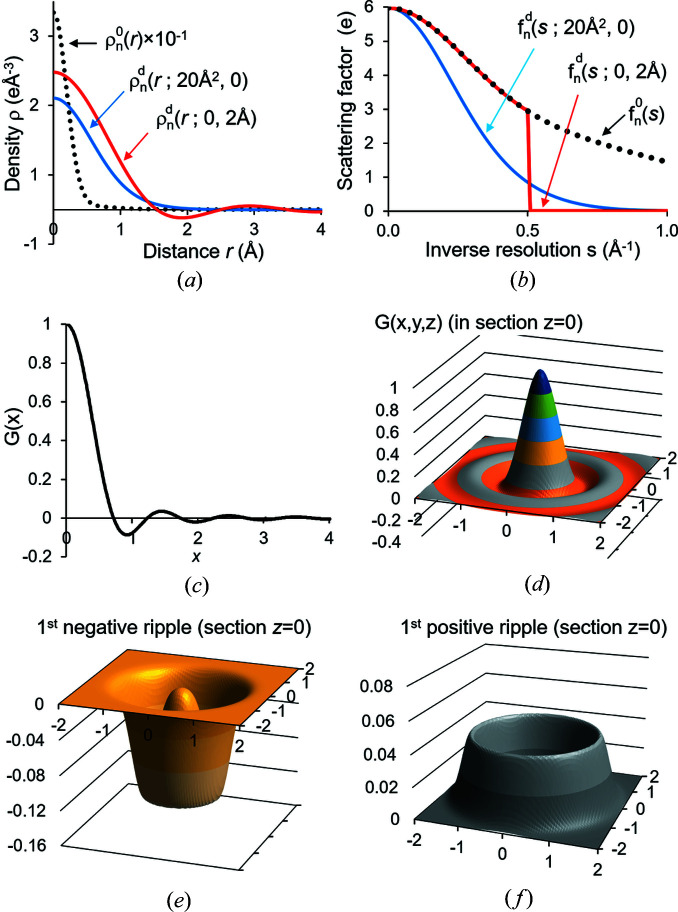
Atomic images and Fourier ripples. (*a*) Electron-density distributions for an immobile carbon atom and its images affected by disorder and resolution cutoff. (*b*) Corresponding scattering functions [Fourier transform of the functions shown in (*a*)]. (*c*) Radial part of the interference function *G*(**x**) and (*d*) this function in the two-dimensional section *z* = 0. Approximation to (*e*) the first negative and (*f*) the first positive ripples of *G*(**x**) by the weighted functions Ω(**x**; μ, ν) according to Table 1 ▸. Two-dimensional section *z* = 0 is shown.

The contributions of atoms to the density ρ(**r**) are usually described by spherically symmetric analytic functions 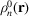 , their atomic densities; here *n* is the consecutive atomic number. A limited-resolution map ρ^*d*^(**r**) of the density ρ(**r**) can also be seen as the sum of atomic contributions 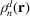 , this time atomic images, which should reproduce respective map distortions. While the atomic positional disorder blurs atomic densities, the resolution cut-off, alongside this, generates Fourier ripples in the resulting image [Figs. 2 ▸(*a*) and 2 ▸(*b*)]. The ripples are observed as spherical waves of a slowly decreasing amplitude; they significantly contribute to the map quite a distance from the atomic center [Fig. 1 ▸(*c*)]. Thus, substitution of one type of map distortion by another (Jakobi *et al.*, 2017 ▸) is not fully appropriate. However, both types of distortion can be described by the same mathematical operation of a convolution but with different functions.

### 2.2. Resolution and harmonic disorder

The term ‘resolution’ in structural biology has different meanings (Urzhumtseva *et al.*, 2013 ▸; Afonine, Klaholz *et al.*, 2018 ▸). Traditionally in this field, when an experimental map is represented by a Fourier series, the resolution cut-off for this series is defined as the shortest period of the Fourier harmonics included in the calculation, and is considered the map resolution (*e.g.* Rupp, 2010 ▸); the number of Fourier harmonics eventually missed is supposed to be negligible. The resolution effect on the image can be mathematically described by the convolution

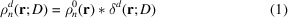

of the atomic density 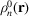 with the spherically symmetric function

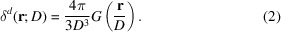

Here 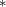 stands for the convolution operation, *D* is the resolution cut-off in the Fourier space, *D* = *d*_high_, and *G*(**x**) is the three-dimensional interference function,

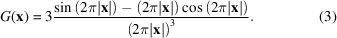

Function *G*(**x**) has a large peak in the origin surrounded by a number of spherically symmetric positive and negative ripples in space (Fig. 2 ▸). Function δ^*d*^(**r**; *D*) may be interpreted as the *D*-resolution image of a virtual immobile point atom, the density of which can be described by the Dirac delta function δ(**r**).

If an experimental map is obtained by an alternative method to that of a single Fourier series, its different regions may reveal patterns typical for maps of a different resolution [Figs. 1 ▸(*a*) and 1 ▸(*b*)]. Such variation of the local resolution is usually illustrated by colored maps and is less commonly available from structural databases.

For the bulk of macromolecular studies, a dynamic and static disorder of atomic positions, individual for each atom, is modeled by a convolution of the respective contributions with a three-dimensional Gaussian function centered in the origin and that is isotropic (isotropic atomic displacement),

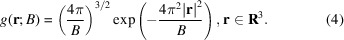

The Gaussian model describes not only the motion of a particular atom around its central position but also the uncertainty of this position, *i.e.* that between numerous copies of the same atom over the sample or different parts of a given sample. An important feature of the Gaussian distribution is that its convolution with other Gaussian functions does not change its form but simply modifies the value of its parameter

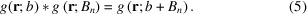

In particular, the density of a Gaussian atom

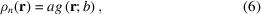

with an isotropic positional disorder described by the atomic displacement parameter *B_n_* can be expressed by

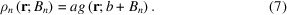

Since the convolutions are commutative, we can combine the two principal sources of the image distortion, (1) and (5), in the most convenient order. The obstacle is an absence of an analytic expression for a convolution of the interference and Gaussian functions.

### 2.3. Shell decomposition of the interference function

**Figure 3:**
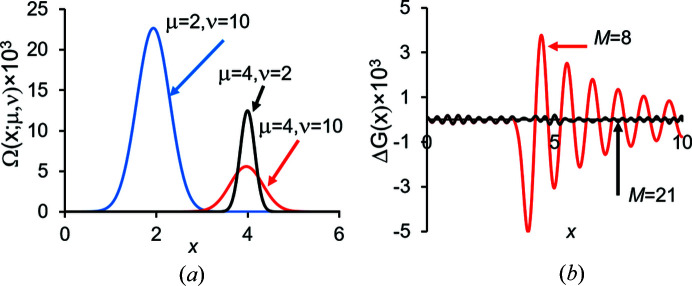
Shell decomposition of the interference function. (*a*) Radial component of the function Ω(**x**; μ, ν) with different μ and ν values indicated on the plot. (*b*) Radial component of the difference between the left and right sides in the decomposition (8) for different numbers *M* of the terms included.

To overcome the latter obstacle, we represent the interference function *G*(**x**) (3) by a linear combination

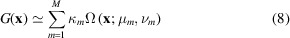

of the terms expressed by the function

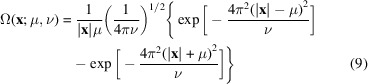

(Fig. 3 ▸). The function Ω(**x**; μ, ν) is the uniform distribution at the spherical surface of the radius μ, blurred by the convolution with the Gaussian function *g*(**x**, ν). It has a convolution property similar to (5), a ‘disorder transferability’:

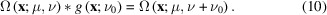

This results in

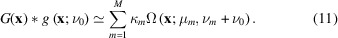

To obtain the explicit form of function Ω(**x**; μ ν), we started from the uniform distribution in a thin spherical shell of radius μ and width Δ, and then applied the convolution theorem and took the limit 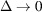 . The Gaussian function *g*(**x**; ν) is a limit case of Ω(**x**; μ, ν) when 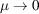 . The sum (8), which we call a shell decomposition, goes beyond the approximation of its central peak by three-dimensional Gaussian functions including also the terms with μ_*m*_ > 0. The values of the parameters μ_*m*_, ν_*m*_ and κ_*m*_ (Table 1 ▸) can be obtained by minimizing the difference between the two sides in (8) and can be modified for other desired accuracy or range of **x** values (Urzhumtseva *et al.*, 2022 ▸).

An important feature of function (9) is that, similar to the Gaussian function, it conserves its form under rescaling,

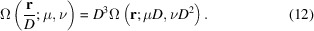




### 2.4. Analytic form of an atomic image

Combining (2), (8) and (12) we obtain the image of an immobile point atom at resolution *D* as

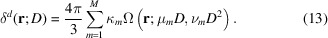

Similarly, the image of a Gaussian atom (6) in the position **r**_*n*_ and possessing the atomic displacement parameter *B_n_* is

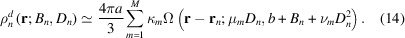

Except for *B_n_*, *D_n_* and the coordinates of the atomic center **r**_*n*_, all to be refined, the other parameters in (14) are external and known in advance.

## 3. Analytic calculation of model maps

In different experimental methods, the contribution 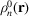 of an immobile atom to the exact density (or equivalently its scattering function) is often approximated by a weighted sum of a few Gaussian functions with the coefficients 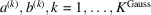 tabulated for each type of atom or ion (*e.g.* Doyle & Turner, 1968 ▸; Agarwal, 1978 ▸; Waasmaier & Kirfel, 1995 ▸; Peng, 1999 ▸; Grosse-Kunstleve *et al.*, 2004 ▸; Brown *et al.*, 2006 ▸). This approximation may vary with the atom environment and may contain positive Gaussians as well as negative ones.

Expressions (9) and (14) allow us to present an atomic model density map, distorted by restricted resolution and positional disorder, in a closed analytical form as

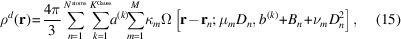

with the function Ω defined in (9), and the values μ_*m*_, ν_*m*_ and κ_*m*_ of the shell decomposition (8) of the interference function universally calculated for the required accuracy (*e.g.* Table 1 ▸). The parameter *B_n_* manifests the uncertainty in the position of the *n*th atom while *D_n_* describes the features of the experimental map in its local environment. These values can be refined together with the atomic position **r**_*n*_, depending on the amount of experimental data available. Besides the calculation of the distorted electron-density map, analytical expression (15) allows us to obtain simple analytic formulae for the derivatives of the map values with respect to all variable parameters. As a consequence, this provides an analytic expression for the gradient that rules the minimization of the discrepancy between the experimental and model maps.

When the variation of the resolution may be neglected either over the whole molecule or locally over a region of interest, the shell decomposition can be used more efficiently. First, one calculates numerically the image 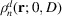 of an immobile atom of every required type at the given resolution *D* as the limited-resolution Fourier transform of the given atomic scattering function. Then the shell decomposition

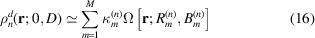

is built directly for each of these few oscillating images. This operation is performed only once, for the chosen resolution *D*. For each atom of the model, the only further adjustment required is increasing the values of the respective parameters 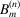 by the atomic *B_n_*. Such an approach can be also used in situations when the multi-Gaussian approximation to these functions is poor or when the scattering functions are defined numerically (*e.g.* Fox *et al.*, 1989 ▸; Brown *et al.*, 2006 ▸; Sorzano *et al.*, 2015 ▸; Murshudov, 2016 ▸). The number *M* of terms to calculate ρ^*d*^(**r**) is reduced roughly by *K* ^Gauss^ times compared with the general scheme, since the summation over Gaussians is no longer required. This accelerates the calculations and may improve iterative refinement procedures due to a smaller number of parameters. Although such a simplified version of the shell decomposition seems to be useful at intermediate stages of model refinement, we expect that the full version with the refinement of *D_n_* parameters would be important at the final stages.

To illustrate the efficiency of our approach we used a test protein model (PDB entry 1zud; Burley *et al.*, 2021 ▸) artificially placed in a unit cell including a single molecule, space group *P*1, and for which we calculated synthetic data and an exact ‘diffraction map’ of 2 Å resolution. Then we calculated a series of Ω-maps (15) with all *D_n_* equal to 2 Å and with *M* that varied from one (the Gaussian peak only) to six. The map calculated, without taking the ripples into account, *M* = 1, was inaccurate in a number of regions whereas inclusion of a few first ripples by an increase to *M* = 5 made the differences with the exact map negligible [Fig. 1 ▸(*c*)]. This proves a need for modeling the Fourier ripples which are ignored in a number of existing methods of map calculation.

To illustrate the possibility of reproducing a map of a prescribed inhomogeneous resolution, we assigned the resolution *D_n_* varying from 2 Å in the model center to 5 Å at its periphery and calculated a respective model map. The central image in Fig. 1 ▸(*b*) shows a fragment of the Ω-map with respective resolutions and calculated in a single run. Indeed, this map is the same as the 2 Å resolution Fourier map in the model center [Fig. 1 ▸(*b*), right] and coincides with the exact 5 Å resolution Fourier map at the periphery [Fig. 1 ▸(*b*), left]. In between, where the local resolution is intermediate, the Ω-map is different from both control Fourier maps. More examples of calculating an Ω-map where its resolution varies from one molecular region to another are given by Urzhumtsev *et al.* (2022 ▸). These examples illustrate a solution of the so-called ‘direct problem’: starting from an atomic model, calculate a map for comparison with an experimental map of an inhomogeneous resolution regardless of the way in which this experimental map has been obtained and the nature of the technique, under the assumption that the atomic scattering functions are known.

Finally, we validated the capacity of (16) to approximate the atomic image for different *B_n_* values. For this goal, we calculated the image of the immobile carbon atom at a resolution of 2 Å and found the coefficients of its shell decomposition. With *M* = 12 terms up to the distance *r* ≤ 8 Å, the relative accuracy with respect to its value in the atomic center 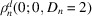 was 10^−4^. For the carbon images with *B_n_* = 10, 20, 30 Å^2^, these coefficients approximated the carbon image (16) with an accuracy close to 5 × 10^−6^ with respect to 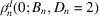 . A similar accuracy was observed for *r* ≤ 4 Å with *M* = 7 terms.

## 4. Results and perspectives

The method developed for calculation of atomic model maps has a number of features crucial for an efficient real-space refinement of atomic models. Such refinement becomes the key to selecting hypothetical protein models suggested by structure prediction methods and to building other model components using various experimental techniques. Our method is aimed at structural studies using cryoEM however manipulating inhomogeneous resolution maps is applicable to other techniques such as X-ray, neutron or electron diffraction. It is based on the hypothesis that principal map distortions are caused by harmonic disorder of the structure and limited resolution which may vary over regions of the map.

First, the method does not require any Fourier transforms as it can reproduce atomic images very accurately (Figs. 1 ▸ and 3 ▸). Second, this method gives an analytic expression for the map values and for their derivatives with respect to all atomic parameters (Urzhumtseva *et al.*, 2022 ▸). Third, the method suggests how to model the heterogeneity of resolution of experimental maps and describes this effect quantitively by the values of the Fourier resolution attributed individually to each atom. Fourth, our method does not only make it trivial to calculate, in a single run, a map with the local resolution that varies from one region to another [Fig. 1 ▸(*b*)], but also to adjust this resolution on-the-fly, to refine it according to the experimental map in the environment of a given atom, and also to deposit it. This ‘inverse problem’ of obtaining parameter values from the given data will be discussed separately.

Above, we considered the basic situation in cryoEM and crystallography. However, the method can be routinely extended to more complicated situations. When increasing the resolution, anisotropic scattering factors or atomic displacement parameters may be required (see, *e.g.* Merritt, 2012 ▸, for a discussion) that currently the suggested decomposition fails to address. However, moving to sub-atomic resolution, macromolecular studies may require taking the atomic environment into consideration and use, for example, multipolar models (Hansen & Coppens, 1978 ▸) both for crystallography (Jelsch *et al.*, 1998 ▸) and for cryoEM macromolecular projects (Yonekura *et al.*, 2015 ▸). It has been shown that instead, one may describe a loss of spherical symmetry in 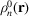 due to density deformation by interatomic scatterers (Afonine *et al.*, 2007 ▸) which allows a routine application of the method described in this work.

The method considers that the scattering function of a given atom or ion are known, in particular for charged atoms (*e.g.* Marques *et al.*, 2019 ▸). Nevertheless, one may dream that, in the eventual case of a large amount of data, it could be possible to start from the scattering functions of neutral atoms and then refine the parameters of these functions against the given map recovering such modified scattering function experimentally.

Even when the decomposition (8) is illustrated by examples in cryoEM, it may be applied to oscillating spherically symmetric functions in other research domains, allowing in particular the calculation of electrostatic potential maps in small and macromolecular charge-density studies (Ghermani *et al.*, 1993 ▸; Muzet *et al.*, 2003 ▸). It may be applied to situations when the atomic density cannot be approximated by a sum of Gaussians, for example, when working with the Coulomb potential. In this case, we can calculate first its image with a relatively small *B*_0_ value, which makes the result ‘Gauss-decomposable’. Then the presented method allows us to obtain images at any resolution and for any *B* ≥ *B*_0_.

The suggested method currently ignores one more source of map errors typical for crystallography: incompleteness of datasets (Urzhumtseva *et al.*, 2013 ▸). In fact, the effect of even a small amount of missing data, distributed randomly, is stronger than expected (Urzhumtsev *et al.*, 2014 ▸). This is the reason why an alternative approach to excluding a test set of data (Brünger, 1992 ▸) has been suggested (Pražnikar & Turk, 2014 ▸). Otherwise, the effect of missed and weighted reflections can be modeled by respective modification of atomic scattering functions when calculating atomic images.

The programs for the suggested decomposition of 3D oscillating functions (Urzhumtseva *et al.*, 2022 ▸) are of a general interest and their scripts, in Python3 and in Fortran77, can be obtained upon request from the authors or from the site https://ibmc.cnrs.fr/en/laboratoire/arn-en/presentation/structures-software-and-websites/.

## Figures and Tables

**Table 1:** Coefficients of the shell decomposition (8) of the function *G*(**x**) obtained for |**x**| ≤ 10, *M* = 21 [see Fig. 3 ▸(*b*)] The central peak of *G*(**x**) is represented by the sum of the Gaussian function 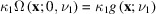 and the correcting function 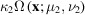 . Each Fourier ripple is represented by one 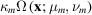 term alternating the sign of κ_*m*_. The maximal error of the approximation is about 2 × 10^−4^ of the maximum of *G*(**x**) equal to 1

*M*	μ	ν	κ	*M*	μ	ν	κ	*M*	μ	ν	κ
1	0.000	10.131	0.693	8	3.492	2.795	0.485	15	6.989	1.670	−0.368
2	0.339	3.216	0.026	9	3.971	2.882	−0.476	16	7.490	1.509	0.356
3	0.873	4.819	−0.797	10	4.471	2.022	0.401	17	7.991	1.369	−0.334
4	1.439	3.622	0.595	11	4.995	1.620	−0.371	18	8.493	1.248	0.326
5	1.979	3.616	−0.599	12	5.504	2.317	0.416	19	8.995	1.146	−0.332
6	2.462	4.143	0.623	13	5.980	2.062	−0.407	20	9.494	1.060	0.333
7	2.953	3.047	−0.534	14	6.490	1.849	0.392	21	9.978	0.811	−0.290
